# Targeted genome engineering in human induced pluripotent stem cells by penetrating TALENs

**DOI:** 10.1186/2045-9769-2-5

**Published:** 2013-06-18

**Authors:** Renli Ru, Yongchao Yao, Songlin Yu, Benpeng Yin, Wanwan Xu, Siting Zhao, Li Qin, Xiaoping Chen

**Affiliations:** 1 Laboratory of Pathogen Biology, State Key Laboratory of Respiratory Disease, Center for Infection and Immunity, Guangzhou Institutes of Biomedicine and Health, Chinese Academy of Sciences, Guangzhou, 510530 China; 2 Guangzhou Science Park, Guangzhou, 510530 P. R. China

**Keywords:** CCR5, HIV-1, Cell-penetrating peptide, TALEN, TAT, Protein delivery, Induced pluripotent stem cells

## Abstract

**Background:**

Zinc-finger nucleases (ZFNs) and transcription activator-like effector nucleases (TALENs) have been successfully used to knock out endogenous genes in stem cell research. However, the deficiencies of current gene-based delivery systems may hamper the clinical application of these nucleases. A new delivery method that can improve the utility of these nucleases is needed.

**Results:**

In this study, we utilized a cell-penetrating peptide-based system for ZFN and TALEN delivery. Functional TAT-ZFN and TAT-TALEN proteins were generated by fusing the cell-penetrating TAT peptide to ZFN and TALEN, respectively. However, TAT-ZFN was difficult to purify in quantities sufficient for analysis in cell culture. Purified TAT-TALEN was able to penetrate cells and disrupt the gene encoding endogenous human chemokine (C-C motif) receptor 5 (CCR5, a co-receptor for HIV-1 entry into cells). Hypothermic treatment greatly enhanced the TAT-TALEN-mediated gene disruption efficiency. A 5% modification rate was observed in human induced pluripotent stem cells (hiPSCs) treated with TAT-TALEN as measured by the Surveyor assay.

**Conclusions:**

TAT-TALEN protein-mediated gene disruption was applicable in hiPSCs and represents a promising technique for gene knockout in stem cells. This new technique may advance the clinical application of TALEN technology.

**Electronic supplementary material:**

The online version of this article (doi:10.1186/2045-9769-2-5) contains supplementary material, which is available to authorized users.

## Background

Zinc-finger nucleases (ZFNs) and transcription activator-like effector nucleases (TALENs) are two types of artificial site-specific nucleases that are generated by fusing the DNA cleavage domain of the FokI restriction endonuclease with a custom-designed DNA biding domain. In ZFNs and TALENs, these DNA-binding domains are the C_2_H_2_ zinc-finger motif [1] and the transcription activator-like effector (TALE) domain respectively [2]. Because the FokI nuclease domain functions as a dimer, ZFNs and TALENs are typically used in pairs. These chimeric nucleases induce DNA double-strand breaks at defined sites in living cells that can be repaired via homology-directed repair (HDR) pathway, which utilizes a homologous donor DNA, or imprecise nonhomologous end joining (NHEJ) pathway [3]. These engineered nucleases have been widely used to modify endogenous genes in human stem cells [4–7]. However, gene-based delivery systems are problematic. In particular, the use of viral vectors entails the risk of insertional mutagenesis [8], whereas plasmid DNA has cell-type restrictions and cytotoxicity.

Cell-penetrating peptides (CPPs), also known as protein transduction domain (PTD) peptides, have been used to deliver a variety of cargoes (including drug molecules, nucleic acids, liposome nanoparticles and large proteins) into cells both *in vivo* and *in vitro* and have therefore shown great promise as therapeutic delivery mechanisms for the treatment of several diseases [9]. The CPP delivery system has various advantages including applicability to all cell types, high transduction efficiency and controlled administration. Among the CPPs, TAT (YGRKKRRQRRR) is the most widely investigated and widely used peptide. TAT is a short peptide consisting of 11 amino acids from the human immunodeficiency virus-1 (HIV-1) TAT protein and is rich in arginine and lysine; therefore, TAT is highly positively charged and hydrophilic [10]. Since the first use of TAT as a delivery vehicle for introducing molecules into cells in the late 1990s, numerous studies have reported the use of TAT for the delivery of various biomolecules (especially proteins) into cells in the form of TAT fusion proteins or TAT-protein conjugates [11]. Use of TAT as a delivery vehicle has been considered as one of the most promising gene-free strategies.

In this study, we designed TAT-ZFN and TAT-TALEN fusion proteins by in-frame cloning. We succeeded in purifying functional TAT-TALEN proteins, and demonstrated their cell penetrating properties. When incubated with living cells, TAT-TALENs efficiently disrupted the endogenous *CCR5* gene under hypothermic conditions in a dose-dependent manner and we observed a disruption efficiency of up to 5% in human induced pluripotent stem cells (hiPSCs).

## Results

### Purification and activity testing of TAT-ZFNs and TAT-TALENs

**Figure 1:**
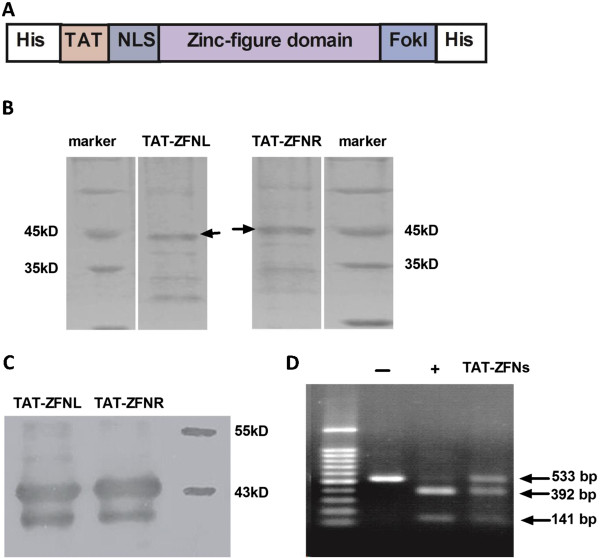
**Purification of TAT-ZFNs.** (**A**) Schematic diagram of the TAT-ZFN construct. (**B**) SDS-PAGE analysis of TAT-ZFNs purified under native conditions. (**C**) Western blot analysis of expressed TAT-ZFNs using an anti-His antibody. (**D**) *In vitro* activity testing of TAT-ZFNs purified under native conditions. The negative control is indicated by “-”. The recognition sequence of Hpy188III is located within the ZFN recognition sequence; therefore, Hpy188III was used as the positive control (+).

The structure of the TAT-ZFN construct is shown in Figure 1A. Soluble proteins were purified as described in Methods. TAT-ZFN purification was greatly limited by its low protein expression level and low binding affinity; thus, only a small amount of TAT-ZFN could be obtained (Figure 1B). Because the purification tag could be detected by anti-His antibodies (Figure 1C), we speculated that the His tag was likely to be hidden by the tertiary structure of the native protein. Nevertheless, TAT-ZFNs showed specific nuclease activity *in vitro* (Figure 1D).

**Figure 2:**
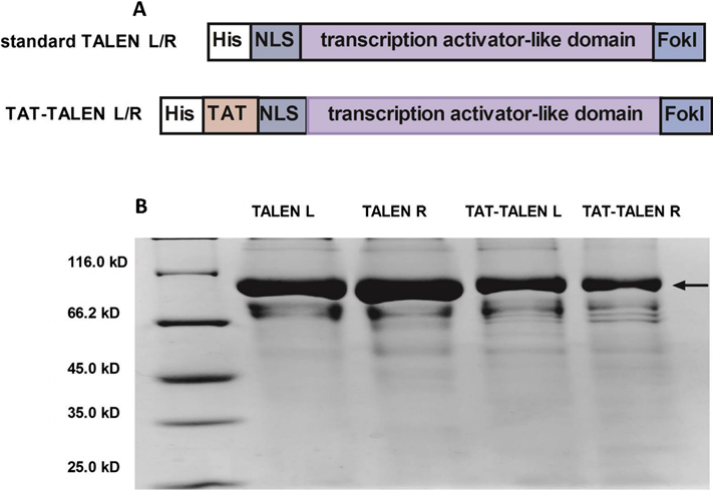
**Purification of standard TALENs and TAT-TALENs.** (**A**) Schematic diagram of standard TALENs and TAT-TALENs. (**B**) SDS-PAGE analysis of four TALEN proteins stained with Coomassie blue after purification.

**Figure 3:**
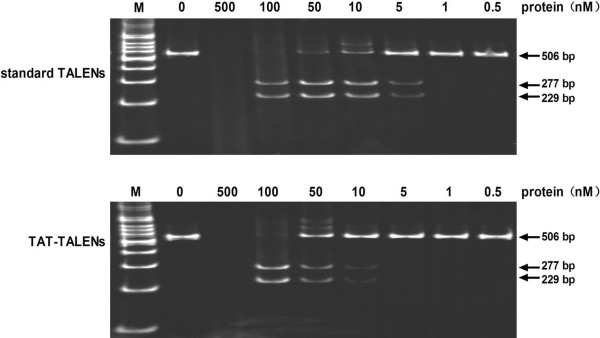
**Activity testing of purified standard TALENs and TAT-TALENs.** Specific activity was determined based on the presence of two cleavage products with lengths of 229 bp and 277 bp.

No problems were encountered with TAT-TALEN purification. Standard TALEN was also constructed and purified for use as a control in subsequent experiments. The structure of standard TALEN and TAT-TALEN is illustrated in Figure 2A. The purified proteins were visualized using sodium dodecyl sulfate polyacrylamide gel electrophoresis (SDS-PAGE) with Coomassie Brilliant Blue staining (Figure 2B). The *in vitro* DNA cleavage assay demonstrated that both purified standard TALEN and TAT-TALEN were functional (Figure 3). Notably, a high TALEN concentration resulted in non-specific DNA cleavage.

### Transduction of proteins into living cells

**Figure 4:**
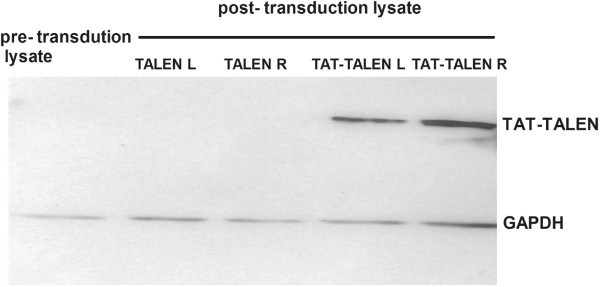
**Western blot analysis of lysate from the treated HeLa cells using an anti-FLAG antibody.** HeLa cells were incubated with TALEN for 1 hour at 37°C and subsequently washed three times with 1 mg/ml heparin-PBS to remove proteins bound to the cell surface before lysis.

To evaluate the ability of the purified proteins to penetrate cells, HeLa cells were treated with 1.5 μM TALEN proteins for 1 hour at 37°C and subsequently washed with 1 mg/ml heparin to remove proteins bound to the cell surface. Immunoblotting of the treated HeLa lysate showed that TALEN itself could not penetrate the cells; however, the 11-amino acid TAT was able to deliver the 110 kD TALEN into the cells (Figure 4).

### *CCR5* disruption in HeLa cells and hiPSCs

**Figure 5:**
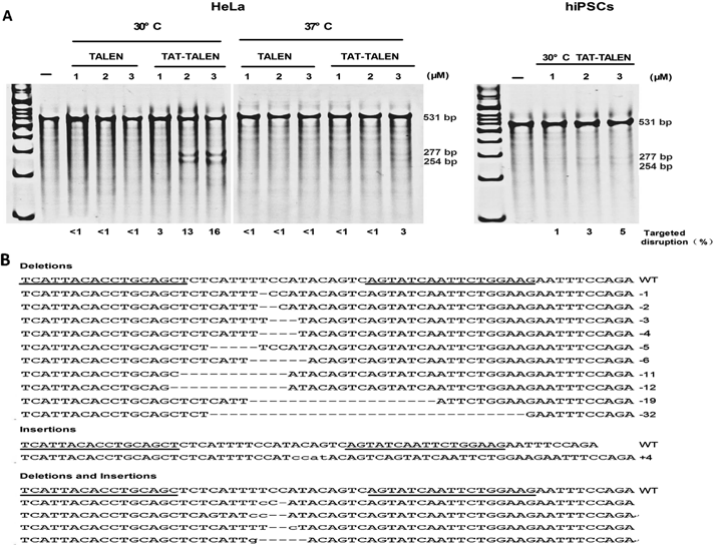
**Disruption of endogenous genes by direct delivery of TALEN proteins.** (**A**) Frequency of endogenous *CCR5* gene disruption in HeLa cells and hiPSCs subjected to three consecutive treatments with TALEN proteins as determined by the Surveyor assay. Surveyor nuclease cleavage at mismatches produces products of 254 bp and 277 bp. (**B**) Representative sequence analysis of the *CCR5* locus in HeLa cells after three consecutive treatments with 3 μM TAT-TALEN proteins under hypothermic conditions. Multiple deletions (dashed) and insertions (lowercase) are aligned with the cleavage site (wild type [WT]). Underlines highlight binding sites for TALEN L and TALEN R.

HeLa cells were subjected to treatment with TALENs at 37°C for 1 hour and then continuously cultured at 37°C for 24 hours before the next treatment. After three treatment cycles, a Surveyor nuclease assay was performed to evaluate gene disruption. TAT-TALENs disrupted *CCR5* at 37°C in a dose-dependent manner, whereas standard TALENs exerted no effects on *CCR5* disruption. As shown in Figure 5A, HeLa cells subjected to three consecutive treatments with 3 μM TAT-TALEN proteins showed a modification frequency of 3% as measured by the Surveyor assay. This modification frequency increased to 16% when the cells treated with TAT-TALEN were incubated at 30°C. Standard TALENs still did not disrupt *CCR5* gene under hypothermic conditions. To verify the reliability of the Surveyor assay, TAT-TALEN mediated *CCR5* disruption was confirmed by sequence analysis of cloned *CCR5* alleles amplified from treated HeLa cells (Figure 5B).

To explore the potential of this method for practical application, we evaluated the effects of *CCR5* gene disruption via TAT-TALEN proteins in hiPSCs. Under hypothermic conditions, hiPSCs subjected to three consecutive treatments with 3 μM TAT-TALEN proteins showed gene disruption frequencies of up to 5% (Figure 5A). It is unknown whether cell membrane composition or endocytosis capabilities could explain the observed differences between HeLa cells and hiPSCs with regard to *CCR5* gene disruption frequency.

### TAT-TALEN cytotoxicity analysis

**Figure 6:**
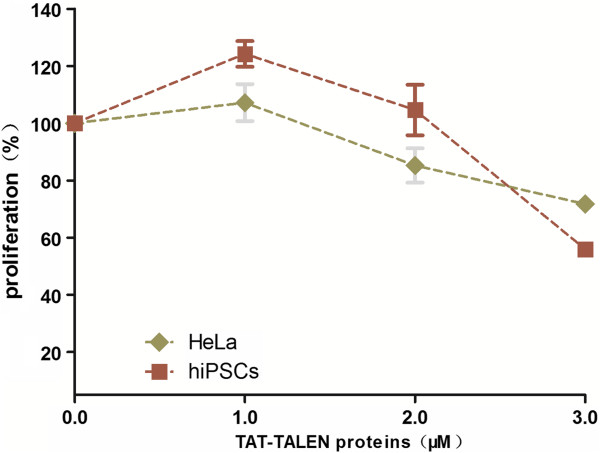
**Proliferation of HeLa cells and iPSCs subjected to three consecutive treatments with various concentrations of TAT-TALEN proteins.** Values are normalized to mock treated cells. Error bars, s.d. (n = 3).

To examine the cytotoxicity of TAT-TALENs, the proliferation of HeLa cells and hiPSCs treated with TAT-TALENs was measured. We observed a decrease in the proliferation of each cell type after three consecutive treatments with TAT-TALENs (Figure 6). Cytotoxicity was also dose-dependent, suggesting that consecutive low doses might be required to minimize potential toxic effects.

## Discussion

Targeted genomic engineering in stem cells at desired endogenous gene loci by ZFNs or TALENs has significant therapeutic implications [4]. The correction of disease-causing mutations in patient-derived stem cells is a goal of curative regenerative medicine. The disruption of a normal gene in stem cells can also be beneficial for the treatment of certain diseases. CCR5 is a major co-receptor for HIV-1 that is present on the surface of target cells [12]. Currently, autologous transplantation of CCR5-deficient human hematopoietic stem cells (hHSCs) has been a promising strategy for acquired immunodeficiency syndrome (AIDS) treatment [13–17]. Notably, HIV-1 can establish latent viral reservoirs in hHSCs [18] prior to genome engineering; thus, caution must be exercised when using these cells. Our lab has successfully disrupted the *CCR5* locus of hiPSCs using ZFNs and demonstrated that these *CCR5*-disrupted hiPSCs can differentiate into CD34+ cells with multipotency *in vitro*[19]. This work contributes to the advancement of patient-specific therapies with genetically modified hiPSCs produced from the healthy tissues of HIV-infected patients. However, some problems such as safety concerns regarding the gene-based delivery method of ZFNs and TALENs need to be addressed [8, 20]. In addition to mRNA delivery system, our TAT-TALEN protein provides an alternative to eliminate the risk of insertional mutagenesis and expands the range of cell types that the nucleases can be applied to modify.

The concept of CPP-ZFN proteins has been proposed previously [21, 22]. However, we failed to utilize TAT-ZFN to modify the endogenous gene because of a purification problem. Recently, a study by Gaj and colleagues demonstrated the intrinsic cell-penetrating capabilities of standard ZFN proteins and their ability to successfully modify *CCR5* in living cells [23]. Compared with ZFNs, TALENs are more mutagenic [24] and more specific [25]; additionally, the design and assemble of TALENs is very simple and straightforward [26, 27]. In all these aspects, TALENs are superior to ZFNs.

We utilized TAT-TALEN to successfully disrupt endogenous *CCR5* in HeLa cells and hiPSCs. It has been reported that hypothermia treatment improves ZFN-driven [28] and TALEN-driven [29] gene disruption in human cells transfected with eukaryotic expression vectors. We found that hypothermic treatment was also effective in cells transduced with TAT-TALENs. A high TALEN concentration may lead to an increase in off-target cleavage events in cells; cells treated with excessive amounts of TAT-TALENs are likely to die as a result of non-specific genome cleavage. This effect may account for the moderate cytotoxicity observed in this experiment. In our study, TAT was fused to the N-terminus of TALEN. It is unknown whether shifting the TAT peptide to the C- terminus of TALEN or using other CPPs such as polyarginine could further enhance protein penetration efficiency; however, similar adjustments need to be considered in future studies.

In summary, we generated functional TAT-TALEN proteins that were capable of penetrating cells. These constructs were used to efficiently disrupt *CCR5* in HeLa cells and hiPSCs. Combined with our previous work, this gene-free delivery system has great potential for the clinical application of *CCR5*-disrupted hiPSCs. In addition, this new TALEN delivery method may be applied to the gene therapy for several monogenic diseases as well, a circumstance in which a DNA donor template is needed.

## Methods

### Construction of prokaryotic expression plasmids

ZFN mammalian expression vectors were previously constructed in our lab [19]. TALEN mammalian expression vectors were constructed as described [26, 29]. The corresponding prokaryotic expression plasmids were constructed as follows: encoding sequences of ZFN and TALEN were cloned from their mammalian expression vectors. TAT-encoding sequences were introduced by primer design. All primer sequences are provided in Additional file 1: Table S1. The PCR products were digested with NdeI and BamHI for (ZFN) or NdeI and HindIII (for TALEN), and the digested products were inserted into pET28a (Novagen) to yield the following six plasmids: pET.TAT-ZFNL, pET.TAT-ZFNR, pET.TAT-TALENL, pET.TAT-TALENR, pET.TALENL, pET.TALENR. L and R stand for left and right, respectively. Left ZFN (or TALEN) and right ZFN (or TALEN) constitute a functional ZFN (or TALEN) pair. Proper construction of each expression cassette was verified by sequence analysis. The amino acid sequences of each protein are provided in Additional file 1: Table S2.

### Expression and purification of fusion proteins

Recombinant expression plasmids were transformed into competent *Escherichia-coli* BL21 (DE3) cells. LB medium (40 ml) supplemented with 100 μg/ml kanamycin was inoculated with a single colony and incubated overnight at 37°C with shaking. On the following day, the overnight culture was diluted 50-fold into fresh LB medium supplemented with 100 μg/ml kanamycin and incubated at 37°C with shaking until the optical density at 600 nm (OD_600_) reached 0.5. The culture was then incubated at 16°C for 1 hour. Protein synthesis was induced by the addition of isopropyl β-d-1-thiogalactopyranoside (IPTG) to a final concentration of 0.7 mM, and the culture was grown overnight at 16°C with shaking. The cells were harvested and lysed five times using a French press (JN-3000 PLUS, JNBIO) at 10,000 psi in lysis buffer (25 mM Tris–HCl, pH 8.0, 300 mM NaCl, 5% V/V glycerol) at 4°C. After the cell debris was removed by centrifugation at 20,000 g for 30 minutes, TALEN proteins in the soluble fraction were purified using Ni-NTA agarose resin (QIAGEN) and eluted with elution buffer (lysis buffer with 500 mM imidazole, pH 8.0). For TAT-ZFN expression and purification, 100 μM ZnCl_2_ was added to all solutions.

Each protein was desalted using PD-10 columns (GE Healthcare) into PBS (pH 7.4) with 20% glycerol and sterilized by filtration through a 0.22 μM filter. The protein was then aliquoted and stored at −80°C. Protein purity was assessed by SDS-PAGE, and the total protein concentration was determined using a BCA protein assay kit (Beyotime, China). The total protein concentrations of standard TALEN and TAT-TALEN were between 0.9 and 1.2 mg/ml and between 0.3 and 0.5 mg/ml, respectively.

#### In vitro activity of purified proteins

Because the recognition sites of ZFN and TALEN are distinct from each other, *CCR5* loci were amplified separately from the human genomic DNA with different primer pairs (Primer Pair 1 for ZFNs and Primer Pair 2 for TALENs). The substrate DNA was incubated with ZFN or TALEN in NEB buffer 4 for 1 hour at 37°C [30]. Cleavage products were separated by agarose gel electrophoresis (1.5%) or polyacrylamide gel electrophoresis (8%) in 1 × TBE buffer and stained with ethidium bromide solution.

### Cell culture

HeLa cells were seeded onto 48-well plates at a density of 5 × 10^4^ cells per well and cultured in a humidified atmosphere containing 5% CO_2_ at 37°C. Cells were maintained in high-glucose DMEM (HyClone) containing 10% fetal bovine serum (FBS) and 1% (v/v) penicillin-streptomycin.

hiPSCs were induced from the urine cells of a healthy individual using episomal vectors. Initial populations were seeded onto 48-well plates at the proper density and grown until cell confluence reached 60-70%. These cells were maintained in a feeder-free culture system using mTesR1 (Stem Cell Technologies, Vancouver, BC, Canada) and Matrigel (BD Bioscience, Bedford, MA) with 1% (v/v) penicillin-streptomycin.

### Protein treatments

TALEN proteins, including standard TALENs and TAT-TALENs were prepared for treatment as follows: TALEN proteins were diluted into serum-free high-glucose DMEM. The cells were washed with serum-free medium and incubated with TALEN proteins for 1 hour at 37°C. After the initial treatment, the cells were washed and maintained at 37°C or shifted to 30°C for 24 hours before the next treatment. This process was repeated three times over four consecutive days.

### Western blot analysis to determine protein internalization

HeLa cells were treated with either 1.5 μM TALEN or 1.5 μM TAT-TALEN for 1 hour at 37°C and then washed three times with 1 mg/ml heparin-PBS to remove proteins bound to the cell surface. After trypsinization, the cells were collected and lysed in RIPA lysis buffer (Beyotime) and subsequently boiled in the presence of loading buffer (50 mM Tris–HCl, pH 6.8, 100 mM dithiothreitol, 2% sodium dodecyl sulfate (SDS), 20% glycerol and 0.2 mg/mL bromophenol blue) for 10 minutes and subjected to electrophoresis using 10% SDS-PAGE. The gel was transferred to a polyvinylidene difluoride membranes using a Trans-blot SD Semi-Dry Electrophoretic Transfer Cell (Bio-Rad Laboratories, Hercules, CA). TALENs were verified using monoclonal anti-FLAG M2 antibodies (Sigma, 1:1000 dilution) and secondary HRP-labeled goat anti-mouse antibodies (Sigma, 1:5000 dilution). The internal loading control, glyceraldehyde-3-phosphate dehydrogenase (GAPDH), was detected with peroxidase-conjugated anti-GAPDH antibody. The western blots were developed with DAB substrate according to the manufacturer’s instructions.

### Surveyor nuclease assay

Genomic DNA was extracted from TALEN-treated cells with a DNeasy Blood and Tissue Kit (QIAGEN). The *CCR5* locus was amplified by nested PCR, and the sequences of the outer and inner primers are provided in Additional file 1: Table S1. After amplification of the *CCR5* locus using Platinum® Taq DNA Polymerase (Invitrogen), a Surveyor nuclease assay was performed following the instructions included with the SURVEYOR Mutation Detection Kit (Transgenomic). Analysis of the gene disruption frequency was performed as described [31]. The gel image was processed with the ImageJ software.

### Cell proliferation assay

HeLa cells and hiPSCs were seeded onto 48-well plates at the optimal densities. The cells were then treated with TAT-TALEN proteins as described above. Cytotoxicity assays were conducted using the Cell Counting Kit-8 (Dojindo Molecular Technologies, Inc.) according to the manufacturer’s instructions.

### Sequence analysis

Genomic DNA from TAT-TALEN-treated HeLa cells was isolated with the DNeasy Blood and Tissue Kit (QIAGEN). The *CCR5* locus was amplified by PCR with the primers BamHI-5’hCCR5 and HindIII-3’hCCR5. Subsequently, the PCR products were cloned into PUC19 plasmids using the BamHI and HindIII restriction sites. *CCR5* disruption was confirmed by sequence analysis of individual cloned transformants.

### Ethical approval

This experiments was approved by the Human Subject Research Ethics Committee (IRB), Guangzhou Institutes of Biomedicine and Health, Chinese Academy of Sciences with the reference number: GIBH-IRB02-2009002.

## Electronic supplementary material

Additional file 1: Table S1.: The sequences of primers used in this study. **Table S2.** The amino acid sequences of the ZFN and TALEN proteins used in this study. (PDF 118 KB)
