# Supplementary material for: Targeted genome engineering in human induced pluripotent stem cells by penetrating TALENs
Source: Cell Regen. 2013 Jun 18;2:5. doi: 10.1186/2045-9769-2-5 (PMC4230761; doi:10.1186/2045-9769-2-5)
Supplement: Supplementary file 1 — Additional file 1: Table S1.: The sequences of primers used in this study. Table S2. The amino acid sequences of the ZFN and TALEN proteins used in this study. (PDF 118 KB) [file 13619_2013_13_MOESM1_ESM.pdf]

Supplementary Table 1. The sequences of primers used in this study

TAT-ZFN L	F： 5’-TCGAGCCATATGTACGGTCGTAAAAAACGTCGTCAGCGTCG TCGTATGGCACCAAAAAAGAAACG-3’
	R： ACGCTCGAGGAAGTTAATCTCGCCATTGTTAAAC
TAT-ZFN R	F： 5’-TCGAGCCATATGTACGGTCGTAAAAAACGTCGTCAGCGTCG TCGTATGGCTCCCAAGAAAAAGAG-3’
	R： same with TAT-ZFN L
TAT-TALEN L	F： 5’-GGAATTCCATATGTACGGTCGTAAAAAACGTCGTCAGCGTCG TCGTATGGCTCCAAAGAAGAAGCG-3’
	R： 5’-CCCAAGCTTTTAAAAGTTTATCTCGCCGTTAT-3’
TAT-TALEN R	F： same with TAT-TALEN L
	R： same with TAT-TALEN L
standard TALEN L	F： 5’-GGAATTCCATATGGCTCCAAAGAAGAAGCG-3’
	R： same with TAT-TALEN L
standard TALEN R	F： same with standard TALEN L
	R： same with TAT-TALEN L
Primer Pair 1	F： 5’-CAGAGCCAAGCTCTCCATCTAG-3’
	R： 5’-GTTGACACATTGTATTTCCAAAG-3’
Primer Pair 2	F： 5’-TCTATTTTATAGGCTTCTTCTCTGG-3’
	R： 5’-CAACCTGTTAGAGCTACTGCAA-3’
Outer primers	F： 5’-AGTGTCAAGTCCAACCTATGAC-3
	R： 5’-GGATCGGGTGTAAACTGAAC-3’
Inner primers	F： 5’- ACAATGTGTCAACTCTTGACAG-3
	R： same with Primer pairs 2 (R)
BamHI-5’hCCR 5	5’-CGCGGATCCACAATGTGTCAACTCTTGACAG-3’
HindIII-3’hCCR 5	5’-CCCAAGCTTCAACCTGTTAGAGCTACTGCAA-3’

F:Forward primer; R: Reverse primer

Supplementary Table 2. The amino acid sequences of the ZFN and TALEN proteins used in this study

TAT-ZFNL

MGSSHHHHHHSSGLVPRGSHMYGRKKRRQRRRMAPKKKRKVGIHGVPAAMAERPFQC
RICMRNFSDRSNLSRHIRTHTGEKPFACDICGRKFAISSNLNSHTKIHTGSQKPFQCRICMR
NFSRSDNLARHIRTHTGEKPFACDICGRKFATSGNLTRHTKIHLRGSQLVKSELEEKKSEL
RHKLKYVPHEYIELIEIARNSTQDRILEMKVMEFFMKVYGYRGKHLGGSRKPDGAIYTVG
SPIDYGVIVDTKAYSGGYNLPIGQADEMQRYVEENQTRNKHINPNEWWKVYPSSVTEFK
FLFVSGHFKGNYKAQLTRLNHITNCNGAVLSVEELLIGGEMIKAGTLTLEEVRRKFNNGEI
NFLEHHHHHH

TAT-ZFNR

MGSSHHHHHHSSGLVPRGSHMYGRKKRRQRRRMAPKKKRKVGIHGVPAAMAERPFQC
RICMRNFSRSDNLSVHIRTHTGEKPFACDICGRKFAQKINLQVHTKIHTGEKPFQCRICMR
NFSRSDVLSEHIRTHTGEKPFACDICGRKFAQRNHRTTHTKIHLRGSQLVKSELEEKKSEL
RHKLKYVPHEYIELIEIARNSTQDRILEMKVMEFFMKVYGYRGKHLGGSRKPDGAIYTVG
SPIDYGVIVDTKAYSGGYNLPIGQADEMQRYVEENQTRNKHINPNEWWKVYPSSVTEFK
FLFVSGHFKGNYKAQLTRLNHITNCNGAVLSVEELLIGGEMIKAGTLTLEEVRRKFNNGEI
NFLEHHHHHH

TALENL

MGSSHHHHHHSSGLVPRGSHMAPKKKRKVDYKDHDGDYKDHDIDYKDDDDKGTVDLR
TLGYSQQQQEKIKPKVRSTVAQHHEALVGHGFTHAHIVALSQHPAALGTVAVKYQDMIAA
LPEATHEAIVGVGKQWSGARALEALLTVAGELRGPPLQLDTGQLLKIAKRGGVTAVEAVH
AWRNALTGAPLNLTPDQVVAIASNGGGKQALETVQRLLPVLCQDHGLTPDQVVAIASHDG
GKQALETVQRLLPVLCQDHGLTPDQVVAIASNIGGKQALETVQRLLPVLCQDHGLTPDQV
VAIASNGGGKQALETVQRLLPVLCQDHGLTPDQVVAIASNGGGKQALETVQRLLPVLCQ
DHGLTPDQVVAIASNIGGKQALETVQRLLPVLCQDHGLTPDQVVAIASHDGGKQALETVQ
RLLPVLCQDHGLTPDQVVAIASNIGGKQALETVQRLLPVLCQDHGLTPDQVVAIASHDGG
KQALETVQRLLPVLCQDHGLTPDQVVAIASHDGGKQALETVQRLLPVLCQDHGLTPDQV
VAIASNGGGKQALETVQRLLPVLCQDHGLTPDQVVAIASNNGGKQALETVQRLLPVLCQ
DHGLTPDQVVAIASHDGGKQALETVQRLLPVLCQDHGLTPDQVVAIASNIGGKQALETVQ
RLLPVLCQDHGLTPDQVVAIASNNGGKQALETVQRLLPVLCQDHGLTPDQVVAIASHDGG
KQALETVQRLLPVLCQDHGLTPDQVVAIASNGGGKQALESIVAQLSRPDPALAALTNDHLV
ALACLGGRPALDAVKKGLPHAPALIKRTNRRIPERTSHRVAGSQLVKSELEEKKSELRHKL
KYVPHEYIELIEIARNPTQDRILEMKVMEFFMKVYGYRGEHLGGSRKPDGAIYTVGSPIDY
GVIVDTKAYSGGYNLPIGQADAMQSYVEENQTRNKHINPNEWWKVYPSSVTEFKFLFVS
GHFKGNYKAQLTRLNHITNCNGAVLSVEELLIGGEMIKAGTLTLEEVRRKFNNGEINF

TALENR

MGSSHHHHHHSSGLVPRGSHMAPKKKRKVDYKDHDGDYKDHDIDYKDDDDKGTVDLR
TLGYSQQQQEKIKPKVRSTVAQHHEALVGHGFTHAHIVALSQHPAALGTVAVKYQDMIAA

LPEATHEAIVGVGKQWSGARALEALLTVAGELRGPPLQLDTGQLLKIAKRGGVTAVEAVH
AWRNALTGAPLNLTPDQVVAIASHDGGKQALETVQRLLPVLCQDHGLTPDQVVAIASNGG
GKQALETVQRLLPVLCQDHGLTPDQVVAIASNGGGKQALETVQRLLPVLCQDHGLTPDQ
VVAIASHDGGKQALETVQRLLPVLCQDHGLTPDQVVAIASHDGGKQALETVQRLLPVLC
QDHGLTPDQVVAIASNIGGKQALETVQRLLPVLCQDHGLTPDQVVAIASNNGGKQALETV
QRLLPVLCQDHGLTPDQVVAIASNIGGKQALETVQRLLPVLCQDHGLTPDQVVAIASNIGG
KQALETVQRLLPVLCQDHGLTPDQVVAIASNGGGKQALETVQRLLPVLCQDHGLTPDQV
VAIASNGGGKQALETVQRLLPVLCQDHGLTPDQVVAIASNNGGKQALETVQRLLPVLCQ
DHGLTPDQVVAIASNIGGKQALETVQRLLPVLCQDHGLTPDQVVAIASNGGGKQALETVQ
RLLPVLCQDHGLTPDQVVAIASNIGGKQALETVQRLLPVLCQDHGLTPDQVVAIASHDGG
KQALETVQRLLPVLCQDHGLTPDQVVAIASNGGGKQALESIVAQLSRPDPALAALTNDHLV
ALACLGGRPALDAVKKGLPHAPALIKRTNRRIPERTSHRVAGSQLVKSELEEKKSELRHKL
KYVPHEYIELIEIARNPTQDRILEMKVMEFFMKVYGYRGEHLGGSRKPDGAIYTVGSPIDY
GVIVDTKAYSGGYNLPIGQADAMQSYVEENQTRNKHINPNEWWKVYPSSVTEFKFLFVS
GHFKGNYKAQLTRLNHITNCNGAVLSVEELLIGGEMIKAGTLTLEEVRRKFNNGEINF

TAT-TALENL

MGSSHHHHHHSSGLVPRGSHMYGRKKRRQRRRMAPKKKRKVDYKDHDGDYKDHDIDY
KDDDDKGTVDLRTLGYSQQQQEKIKPKVRSTVAQHHEALVGHGFTHAHIVALSQHPAAL
GTVAVKYQDMIAALPEATHEAIVGVGKQWSGARALEALLTVAGELRGPPLQLDTGQLLKI
AKRGGVTAVEAVHAWRNALTGAPLNLTPDQVVAIASNGGGKQALETVQRLLPVLCQDHG
LTPDQVVAIASHDGGKQALETVQRLLPVLCQDHGLTPDQVVAIASNIGGKQALETVQRLL
PVLCQDHGLTPDQVVAIASNGGGKQALETVQRLLPVLCQDHGLTPDQVVAIASNGGGKQ
ALETVQRLLPVLCQDHGLTPDQVVAIASNIGGKQALETVQRLLPVLCQDHGLTPDQVVAI
ASHDGGKQALETVQRLLPVLCQDHGLTPDQVVAIASNIGGKQALETVQRLLPVLCQDHGL
TPDQVVAIASHDGGKQALETVQRLLPVLCQDHGLTPDQVVAIASHDGGKQALETVQRLLP
VLCQDHGLTPDQVVAIASNGGGKQALETVQRLLPVLCQDHGLTPDQVVAIASNNGGKQA
LETVQRLLPVLCQDHGLTPDQVVAIASHDGGKQALETVQRLLPVLCQDHGLTPDQVVAIA
SNIGGKQALETVQRLLPVLCQDHGLTPDQVVAIASNNGGKQALETVQRLLPVLCQDHGLT
PDQVVAIASHDGGKQALETVQRLLPVLCQDHGLTPDQVVAIASNGGGKQALESIVAQLSR
PDPALAALTNDHLVALACLGGRPALDAVKKGLPHAPALIKRTNRRIPERTSHRVAGSQLVKS
ELEEKKSELRHKLKYVPHEYIELIEIARNPTQDRILEMKVMEFFMKVYGYRGEHLGGSRK
PDGAIYTVGSPIDYGVIVDTKAYSGGYNLPIGQADAMQSYVEENQTRNKHINPNEWWKV
YPSSVTEFKFLFVSGHFKGNYKAQLTRLNHITNCNGAVLSVEELLIGGEMIKAGTLTLEEV
RRKFNNGEINF

TAT-TALENR

MGSSHHHHHHSSGLVPRGSHMYGRKKRRQRRRMAPKKKRKVDYKDHDGDYKDHDIDY
KDDDDKGTVDLRTLGYSQQQQEKIKPKVRSTVAQHHEALVGHGFTHAHIVALSQHPAAL
GTVAVKYQDMIAALPEATHEAIVGVGKQWSGARALEALLTVAGELRGPPLQLDTGQLLKI
AKRGGVTAVEAVHAWRNALTGAPLNLTPDQVVAIASHDGGKQALETVQRLLPVLCQDHG
LTPDQVVAIASNGGGKQALETVQRLLPVLCQDHGLTPDQVVAIASNGGGKQALETVQRLL
PVLCQDHGLTPDQVVAIASHDGGKQALETVQRLLPVLCQDHGLTPDQVVAIASHDGGKQ
ALETVQRLLPVLCQDHGLTPDQVVAIASNIGGKQALETVQRLLPVLCQDHGLTPDQVVAI

ASNNGGKQALETVQRLLPVLCQDHGLTPDQVVAIASNIGGKQALETVQRLLPVLCQDHGL
TPDQVVAIASNIGGKQALETVQRLLPVLCQDHGLTPDQVVAIASNGGGKQALETVQRLLP
VLCQDHGLTPDQVVAIASNGGGKQALETVQRLLPVLCQDHGLTPDQVVAIASNNGGKQA
LETVQRLLPVLCQDHGLTPDQVVAIASNIGGKQALETVQRLLPVLCQDHGLTPDQVVAIAS
NGGGKQALETVQRLLPVLCQDHGLTPDQVVAIASNIGGKQALETVQRLLPVLCQDHGLTP
DQVVAIASHDGGKQALETVQRLLPVLCQDHGLTPDQVVAIASNGGGKQALESIVAQLSRP
DPALAALTNDHLVALACLGGRPALDAVKKGLPHAPALIKRTNRRIPERTSHRVAGSQLVKS
ELEEKKSELRHKLKYVPHEYIELIEIARNPTQDRILEMKVMEFFMKVYGYRGEHLGGSRK
PDGAIYTVGSPIDYGVIVDTKAYSGGYNLPIGQADAMQSYVEENQTRNKHINPNEWWKV
YPSSVTEFKFLFVSGHFKGNYKAQLTRLNHITNCNGAVLSVEELLIGGEMIKAGTLTLEEV
RRKFNNGEINF

The TAT peptide is highlighted red.
